# Subcutaneous Immunoglobulin in Chronic Inflammatory Demyelinating Polyneuropathy: A Historical Perspective

**DOI:** 10.3390/jcm12226961

**Published:** 2023-11-07

**Authors:** Dario Cocito, Erdita Peci, Maria Claudia Torrieri, Marinella Clerico

**Affiliations:** 1 Clinical and Biological Sciences Department, University of Turin, 10043 Orbassano, Italy; 2 Academic Neurology Unit, San Luigi Gonzaga University Hospital, 10043 Orbassano, Italy; mariaclaudia.torrieri@gmail.com; 3 Academic Neurology Unit, San Luigi Gonzaga University Hospital, Clinical and Biological Sciences Department, University of Turin, 10043 Orbassano, Italy; marinella.clerico@unito.it

**Keywords:** chronic inflammatory demyelinating polyneuropathy, therapy, immunoglobulin, intravenous, subcutaneous

## Abstract

The therapeutic administration of subcutaneous immunoglobulin (SCIg) offers various advantages over intravenous immunoglobulin (IVIg). This narrative review examines and compares SCIg versus IVIg in chronic inflammatory demyelinating polyneuropathy (CIDP). SCIg is as effective as IVIg but is better tolerated and easier to administer, as intravenous access is not required. Furthermore, SCIg administration is more convenient and cost-effective than IVIg, enabling flexible treatment scheduling at home and improving patients’ overall quality of life. The availability of highly concentrated immunoglobulin G (IgG) subcutaneous solutions, such as IgPro20, a 20% IgG solution stabilized with L-proline, allows for the administration of larger volumes in a single session, while the parallel development of new technological devices enables the delivery of higher doses over a shorter time. Based on the results of the PATH study, SCIg has become a well-established therapy in CIDP. In addition to discussing the advantages of SCIg, this review summarizes the evolution of SCIg by discussing all the relevant clinical studies which have considered its use in the treatment of CIDP.

## 1. Introduction

Chronic inflammatory demyelinating polyneuropathy (CIDP), an acquired peripheral sensory–motor neuropathy with an assumed autoimmune-mediated pathogenesis [1], is variably characterized by a progressive, relapsing–remitting or monophasic course, differently impacting the everyday activities and quality of life (QoL) of affected patients [2]. CIDP has a worldwide prevalence of between 0.5 and 8.9/100,000 [3,4,5,6,7,8] and is diagnosed through a combination of clinical, electrodiagnostic, and laboratory criteria, recently established by the European Federation of Neurological Societies and Peripheral Nerve Society (EFNS/PNS) [9].

Currently, treatment with immunoglobulins G (IgG) is recommended as first-line therapy by clinical guidelines for chronic dysimmune neuropathies, such as CIDP and multifocal motor neuropathy (MMN) [10], and is administered as either intravenous IgG (IVIg) or subcutaneous IgG (SCIg).

In this review, some of the main aspects of SCIg treatment in CIDP will be described, as well as the differences in this treatment compared with IVIg.

Through searches in MEDLINE, PubMed, EMBASE, BIOSOS, Web of Science, and Cochrane library on 31 March 2023, 185 studies were identified (key words: chronic inflammatory demyelinating polyneuropathy; therapy; immunoglobulin; intravenous; subcutaneous). Fifty-one studies were included.

We acknowledge that a weak point of this narrative review is that due to its structure, it did not allow us to emphasize the levels of evidence supporting the therapies discussed.

## 2. Relevant Sections

### 2.1. Improved Tolerability with SCIg

A meta-analysis that included 138 patients overall from eight studies of SCIg versus IVIg in the treatment of CIDP concluded that the risk of moderate or systemic adverse events (AEs) with SCIg was 28% lower than with IVIg [11]. This meta-analysis did not consider the risk of injection site reactions, which are known to be more common with SCIg than with IVIg [12] but are generally mild, tend to improve with continuing treatment, and do not appear to substantially affect patient treatment satisfaction [11,12].

The less frequent occurrence of moderate or systemic AEs may be accounted for by a difference in the pharmacokinetics of larger-volume intermittent IVIg boluses compared with smaller and more frequently administered SCIg infusions. In fact, bolus infusion with IVIg produces a spike in the concentration of serum immunoglobulin G (IgG), which drops sharply by 30–40% over 48–72 h due to the passage of IgG from the intravascular compartment to the extracellular space. IgG levels then slowly decrease following the first-order kinetics of IgG degradation [13]. On the other hand, after SCIg administration, IgG forms a subcutaneous depot and is slowly absorbed into the lymph and blood over a period of 24–72 h [13]. After 6–12 weeks of repeated once-weekly SCIg infusions at a given dose, IgG concentrations reach a near-steady state, fluctuating within less than 5–10% of the obtained mean IgG concentration [12]. Overall, this results in transient mild-to-moderate localized reactions in contrast to systemic AEs that are more likely to occur with IVIg [14,15,16].

### 2.2. Reductions in Treatment-Related Fluctuations and Wearing-Off with SCIg

Wearing-off and treatment-related fluctuations in patient strength and functional capability are likely due to a progressive decline in IgG concentrations towards the end of an IVIg dosing cycle [17,18]. A 20-week study found that switching from IVIg to SCIg did not change fluctuations in muscle strength, but diminished fluctuations in functional muscle performance were observed [19]. Another 1-year extension study showed that SCIg stabilizes muscle performance and disability in patients with CIDP and suggested that a steady serum IgG concentration is an important factor in preserving muscle strength over time [20]. Of note, the lack of treatment-related fluctuations was the main reason for patients choosing to continue self-administration of SCIg.

### 2.3. Improved Manageability of Administration with SCIg

SCIg administration does not require venous access, nor is there a need for premedication with corticosteroids and antihistamines. These factors reflect, along with the typical pharmacokinetic properties of SCIg, the reduced incidence and severity of SCIg-induced systemic AEs [13]. In addition, the SCIg administration technique is easy to learn, enabling patients to treat themselves independently at home [21,22,23].

### 2.4. SCIgs Are More Cost-Effective

With SCIg administration, there is not always a need for the patient to be hospitalized, to attend a medical examination, or be visited by a nurse, thus lowering the long-term healthcare costs [24]. In a review of 20 studies of SCIg administration in 126 patients with CIDP and 82 with MMN, the overall conclusion was that SCIg was effective, well-tolerated, and cost-effective compared with IVIg; however, most of these studies were case reports or case series [25].

### 2.5. SCIg Therapy: A Historical Perspective

The first account of the therapeutic usage of SCIg dates back to 1952, when Bruton successfully treated a child with what is now known as X-linked agammaglobulinemia (XLA) and recurrent pneumococcal infections by SC administration of human IgG at 3.2 g/month [26]. In 1980, nearly 30 years later, Berger and colleagues were the first to report the administration of SCIg using small pumps that delivered slow SC infusions (1–2 mL/h) in patients with primary antibody deficiency [27]. This method of administration produced little pain or swelling at the infusion site, no systemic reactions, and contributed towards improvements in patient QoL [27]. Between 1980 and 1988, the use of slow SCIg infusions extended to Europe and New Zealand, and in 1981, it was introduced in the Netherlands as a home-based therapy [24].

The first reference to SCIg therapy in CIDP was a case report published in 2006 by Köller and colleagues [28], in which a patient with CIDP was treated for 6 months with SCIg at a weekly dose of 0.1 g/kg, equivalent to a cumulative monthly dose of 0.4 g/kg. The treatment, which was well-tolerated, led to improvements in the Inflammatory Neuropathy Cause and Treatment (INCAT) disability score and the Medical Research Council (MRC) sum score. No local reactions at the injection sites were observed, and the patient reported high satisfaction with the route of administration [28].

The next account of SCIg use in CIDP was a 2008 report of two patients treated with SCIg after 5 and 13 years of previous IVIg therapy [29]. Both patients were receiving mycophenolate mofetil as concomitant immunosuppressive therapy. Apart from mild local skin reactions, the SCIg administration was well-tolerated and led to the stabilization of the disease course, with the patients gaining muscle strength and experiencing less disability [29].

Table 1 summarizes the key studies included in this section regarding the clinical evidence for the efficacy and safety of SCIg for treatment of CIDP.

In 2011, Cocito and colleagues [30] published the results of an Italian prospective longitudinal study of five CIDP patients who had shown clinical improvement after at least 12 months of IVIg, had been clinically stable for at least 3 months, and then switched to SCIg at a monthly dose equivalent to the dose received during IVIg therapy. Muscle strength, disability, grip strength, the incidence of any adverse effects, QoL (tested with the Short-Form (SF)—36 questionnaire [36]), patient treatment satisfaction (using the Modified Life Quality Index (LQI) [37]), and patient preference for route of administration were assessed at baseline, 7–15 days after the last IVIg infusion, and after 6 months of SCIg treatment. Four out of five patients preferred SCIg, and all outcomes showed no significant difference between IVIg and SCIg. The authors concluded that SCIg administration was as efficacious as IVIg in patients who had previously responded to IVIg [30].

In 2013, the first small, double-blind, placebo-controlled, parallel-group, randomized controlled trial (RCT) evaluated the efficacy of switching from IVIg to SCIg maintenance therapy in 30 adult patients with CIDP [31]. After 12 weeks, patients who switched to SCIg experienced a significant increase in isokinetic muscle strength (mean ± standard deviation (SD) change +5.5 ± 9.5%, *p* < 0.05 vs. baseline) compared with an expected decline (mean ± SD change –14.4 ± 20.3%, *p* < 0.05 vs. baseline) in those who received placebo (*p* < 0.01 SCIg vs. placebo). Other key functional measures followed a similar pattern in relation to placebo. SCIg treatment was well-tolerated, with only six patients reporting mild AEs at the injection site. No systemic AEs were recorded, and 70% of the patients preferred SCIg over IVIg maintenance therapy, reporting a more stable condition, increased autonomy, and fewer AEs. The authors concluded that, due to its safety and effectiveness, SCIg treatment is a feasible alternative to IVIg for patients with CIDP [31]. While this initial 12-week study did not provide long-term data on SCIg tolerability and effects on muscle strength, the following year, a 12-month follow-up study of the same group of patients evaluated the longer-term effects of SCIg on muscle strength [20]. In order to preserve a satisfactory clinical response in patients, the SCIg dose was increased by 12%. Overall, muscle strength increased from baseline by 7.2% (*p* = 0.033), with nonsignificant interim improvements observed after 3, 6, and 12 months (5.7%, 8.2%, and 6.8%, respectively); the MRC score increased significantly by 1.7% (*p* = 0.007), whereas other key functional parameters remained unchanged [20]. This study also evaluated the health economic aspects of the switch from IVIg to SCIg, indicating that SCIg is more cost-effective than IVIg, although this result may depend on the national healthcare system [20].

The findings regarding the cost-effectiveness of SCIg versus IVIg were consistent with an Italian study by Lazzaro and colleagues [38], which also considered nonhealthcare costs (e.g., transport and parking, loss of working/school days, and leisure time for patients and caregivers) and reported savings in favor of SCIg of up to EUR 1361 (based on 2013 costs), with IgG being the cost driver for both SCIg and IVIg therapies (94.1% vs. 86.1%, respectively) [38].

To date, very few studies have focused on the various aspects of patient well-being, such as QoL and patient satisfaction, which may be the result of a combination of factors, including the efficacy of Ig therapy, occurrence of AEs, the setting of IgG administration, and discomfort.

A nationwide Italian multicenter prospective observational study [32] reported follow-up data for 66 patients with CIDP who switched from monthly IVIg to weekly SCIg. The primary endpoints of this study were changes in the Overall Neuropathy Limitation Scale (ONLS) score, MRC sum score, and LQI score assessed at the time of switching treatments and at the end of the 16-week follow-up. The ONLS total score significantly improved from a mean ± SD of 4.1 ± 2.8 to 3.1 ± 2.0 points (*p* = 0.018), whereas the MRC score was unchanged, indicating that patients in the SCIg group maintained muscle strength. Scores on the LQI-I subscale (indicating treatment interference in the activities of daily living), LQI-II subscale (indicating problems related to the administration of therapies), and LQI-III subscale (indicating the patient’s perception of the therapeutic setting) significantly improved among patients with CIDP (*p* = 0.016, *p* = 0.021, and *p* = 0.044, respectively) [32]. This study could not show the superiority of one treatment over the other due to the lack of a control group and short follow-up) [32].

In a 2-year retrospective follow-up of their 2014 study, Cocito and colleagues [33] evaluated adherence to therapy, as well as QoL and clinical predictors of long-term disability in 45 patients with CIDP who switched from IVIg to 20% SCIg. Adherence to SCIg treatment decreased to 75.6% at 24 months, but the switch from IVIg to SCIg saw a global improvement in the LQI index, despite it being accompanied by an average increase in IgG consumption of 3.04% [33].

In 2017, the first study to address the effects of SCIg in treatment-naïve CIDP patients was published by Markvardsen and colleagues [35]. In this single-blind cross-over study, 20 CIDP patients were randomized (1:1) to either SCIg 0.4 g/kg/week for 5 weeks or IVIg 0.4 g/kg/day for 5 days [35]. After 10 weeks, patients were switched between treatments and followed for a further 10 weeks. It is worth noting that patients received a higher SCIg dosage than that reported in studies of SCIg maintenance therapy in CIDP [20]. Both SCIg and IVIg treatment improved the muscle strength of treatment-naïve CIDP patients (*p* = 0.0008 vs. baseline) to a similar degree (*p* = 0.80). Overall, the improvements from baseline in combined isokinetic muscle strength (cIKS) were similar (mean ± SD 7.4 ± 14.5%, *p* = 0.0003 with SCIg and 6.9 ± 16.8%, *p* = 0.002 with IVIg), but the time profiles were different, with effects of IVIg on muscle strength peaking earlier than those of SCIg. On the other hand, significant improvements in disability were only observed during SCIg treatment. Data also suggested that IgG plasma concentrations and variation in cIKS were related [35]. The study concluded that the same doses of SCIg and IVIg have similar effects on improving muscle strength in treatment-naïve CIDP patients.

The efficacy of SCIg treatment for CIDP in maintaining muscle strength for up to 1 year, as well as longer term, and in improving disability and QoL has been previously established by several studies [20,31,32,33,39]. The similar efficacy of SCIg to IVIg was also corroborated by a meta-analysis of eight studies of patients with either CIDP or MMN [11]. However, when compared with IVIg, SCIg showed a 28% reduction in the risk of moderate and/or severe AEs (95% confidence interval [CI]: 11–76%) [11].

The first large-scale RCT testing the safety and efficacy of SCIg in patients with CIDP was the PATH study [23]. The trial investigated two different doses of SCIg IgPro20 (Hizentra^®^) for maintenance therapy, examining the rate of relapse or withdrawal from the study (efficacy endpoints). IVIg-dependent patients (*n* = 172) were stabilized on IVIg and then randomized to SCIg IgPro20, at either 0.2 g/kg/week (low dose) or 0.4 g/kg/week (high dose), or placebo for 24 weeks or until relapse or study withdrawal. The proportion of patients who suffered relapses or were withdrawn from the study was significantly greater in the placebo group (63%) compared with the low-dose (39%) or high-dose (33%) SCIg groups (*p* = 0.0007) [23].

SCIg was also well-tolerated; the rates of local and systemic AEs were low. In addition, health-related QoL outcomes were better for both SCIg groups compared with the placebo group. The patients’ preference for weekly SCIg compared with monthly IVIg was explained in terms of increased independence and fewer AEs [23].

A subsequent analysis has evaluated changes in patient-reported outcomes in patients who took part in the PATH study from baseline to last postdose observation, including QoL, treatment satisfaction, and work productivity [40]. This study showed that both SCIg doses effectively stabilized CIDP patients and prevented relapse, were well-tolerated when administered in high volumes, and that patients on a standard IVIg regimen could safely switch to SCIg therapy [40], thus supporting the findings of other smaller studies on the clinical efficacy and safety of weekly SCIg [11].

An open-label, prospective, 48-week extension of the PATH study was conducted to assess the long-term safety (primary objective) and efficacy (secondary objective) of SCIg IgPro20 maintenance therapy in patients with CIDP. In this study, the relapse rate was 10% (*n* = 7/72) with high-dose SCIg and 48% (*n* = 35/73) with low-dose SCIg [41]. Of the seven patients who relapsed during high-dose SCIg therapy, three had a full recovery without further intervention. In addition, 24 of the 26 patients (92%) who relapsed after switching to low-dose SCIg had a complete recovery [41]. At the end of the PATH extension study, 82.4% of patients preferred their current SC treatment, 12.2% preferred IV treatment, and 5.4% had no treatment preference [41]. The most common reasons for preferring SC treatment were perceived greater independence (71.6%), less time required (40.5%), preferable administration frequency (37.8%), and fewer AEs (31.1%) [41].

Despite the increasing number of case reports and clinical trials on the efficacy of SCIg, there remains a lack of clinical guidelines regarding the recommended timing of initiating SCIg therapy in patients with CIDP. A first attempt to address this issue was made by Cirillo and colleagues [34], who evaluated the effect of early initiation of long-term SCIg therapy in CIDP patients. Sixteen treatment-naïve patients who had responded to one cycle of IVIg 0.4 g/kg/day (administered for 5 consecutive days) were switched to SCIg 0.4 g/kg/week for 24 weeks. After switching to SCIg, the MRC and Overall Disability Sum Scores (ODSS) improved significantly at 12 and 24 months (*p* < 0.005), supporting the feasibility of SCIg as an alternative to IVIg in the long-term treatment of CIDP [34]. The early start and long duration of SCIg treatment significantly improved the demyelinating features of nerve conduction and clinical variables, showing correlations between MRC and distal compound muscle action potential amplitude, ODSS, and sensory nerve action potential amplitude and INCAT sensory sum score [34].

As has emerged from the first years of their development, recently, different studies have substantially confirmed the efficacy, safety, and improvement in QoL with SCIg in CIDP therapy [9,42,43,44,45].

## 3. Conclusions and Future Directions

SCIg administration offers advantages over IVIg therapy and appears to be preferred by many patients with CIDP for several reasons. SCIg is mostly associated with transient mild-to-moderate local reactions and fewer nonserious and serious systemic AEs than IVIg. SCIg is associated with reduced severity in end-of-dosing wear-off and results in increasing serum IgG concentrations, which reach nearly constant steady-state levels, with no “peaks-and-troughs” that are typical of IVIg administration. As the subcutaneous route of administration does not require venous access, SCIg can be self-administered at home, which increases flexibility, patient QoL, and cost-effectiveness.

The currently available literature provides robust evidence that SCIg is a safe and effective alternative to IVIg for the maintenance treatment of CIDP, and it also suggests that it is an effective therapeutic option in treatment-naïve CIDP patients.

Another important aspect is the lack of response to both IVIg and SCIg, which has been shown in 35% of the patients [46,47,48,49]. Among the main reasons for this unsuccess are the inadequate diagnosis of CIDP and the presence of antibodies directed against the node and paranode. When the patient positively responds to the therapy, the duration of such a response is extremely variable (10–15 days), and it mainly depends on the Ig clearance. Recently, the FC fragment has been highlighted as a key factor in determining Ig half-life [50]. In the interstitial fluids, the IgG half-life is about 3 weeks long, which is much longer when compared with that of IgA and IgM. The role of the FcRn is to bind the endogenous Igs and to protect them from lysosomal degradation by moving them back to the cellular surface and by bringing them back to the bloodstream [9,50,51]. By doing this, FcRn influences the therapeutic response to the immunoglobulin.

The analysis of the polymorphism of the genes that codify for FcRn [50], together with the analysis of FcRn expression, could be good predictive markers for immunoglobulin therapeutic outcome and for the optimization of their dose and frequency of administration.

## Figures and Tables

**Table 1:** A synopsis of selected studies that demonstrate the efficacy and tolerability of SCIg in patients with CIDP or MMN.

Author, Year Ref.	Study Design	Pathology	SCIg Dosage and Administration	Comparator	Study Duration (wks) ^a^	Effect of SCIg Treatment		
						↑ IgG Levels (Y/N)	Clinical Parameters	SAEs (% pts)
*Switching studies* (*IVIg* → *SCIg*)								
Cocito et al., 2011 [30]	PR, NC	CIDP	Equivalent to prestudy IVIg dose ^b^ (*n* = 5)		24	NR	No clinically significant change vs. baseline	0
Markvardsen et al., 2013 [31]	RCT	CIDP	Equivalent to prestudy IVIg dose ^b^ (*n* = 14)	Placebo (*n* = 15)	12	Y	Improved	0
Markvardsen et al., 2014 [20]	OLE of RCT [31]	CIDP	Equivalent to prestudy IVIg dose ^b^ (*n* = 17)	NA	52	N	Improved	NR
Cocito et al., 2014 [32]	OBS, PR	CIDP (*n* = 66) MMN (*n* = 21)	Equivalent to prestudy IVIg dose ^b^ 16% SCIg (*n* = 6) 20% SCIg (*n* = 81) 1–3 SC infusions/week	NA	17	NR	Improved or maintained vs. baseline	NR
Cocito et al., 2016 [33]	RETRO ^c^	CIDP	20% SCIg equivalent to prestudy IVIg dose (*n* = 45)	Baseline	104	NR	LQI index improved	NR
Van Schaik et al., 2018 [23]	RCT	CIDP	SCIg 0.4 g/kg (*n* = 58) SCIg 0.2 g/kg (*n* = 57)	Placebo (*n* = 57)	24	NR	Improved	3 (SCIg 0.4 g/kg) 5 (SCIg 0.2 g/kg) 2 (placebo)
Cirillo et al., 2018 [34]	OBS, PR	CIDP	IVIg 0.4 g/kg/d for 5 days (1 cycle) → SCIg 0.4 g/kg/week (*n* = 16)	Baseline (pre-Ig treatment)	104	NR	Improved	NR
*Treatment-naïve patients*								
Markvardsen et al., 2017 [35]	RCT, CO	CIDP	SCIg 0.4 g/kg/week for 5 weeks → IVIg 0.4 g/kg/day for 5 days (*n* = 10) IVIg 0.4 g/kg/day for 5 days → SCIg 0.4 g/kg/week for 5 weeks (*n* = 10)	NA	10	Y	Improved	NR

*CIDP* chronic demyelinating polyneuropathy, *CO* crossover, *IVIg* intravenous immunoglobulin, *LQI* Life Quality Index, *MMN* multifocal motor neuropathy, *MPT* manual push technique, *NA* not applicable, *NC* noncomparative, *NR* not reported, *OBS* observational, *OLE* open-label extension, *PR* prospective, *RCT* randomized controlled trial, *RETRO* retrospective, *SAE* serious adverse event, *SCIg* subcutaneous immunoglobulin, *wks* weeks. ^a^ Study duration converted from years, months or days to approximate number of weeks where duration of study/follow-up was not reported as weeks in the source publication. ^b^ Dose equivalency established by study authors. ^c^ Retrospective follow-up study of 2014 study [35].

## Data Availability

Not applicable.
